# Everyday navigation between adaptation and resistance: How young people negotiate their well-being in relation to assigned migrant positions in school

**DOI:** 10.1371/journal.pone.0279762

**Published:** 2023-02-16

**Authors:** Ulrika Lögdberg, Magnus Öhlander, Bo Nilsson

**Affiliations:** 1 School of Health and Welfare, Halmstad University, Halmstad, Sweden; 2 Department of Ethnology, History of Religions and Gender Studies, Stockholm University, Stockholm, Sweden; 3 Department of Culture and Media Studies, Umeå University, Umeå, Sweden; Ilia State University, GEORGIA

## Abstract

Concerning the so-called “refugee crisis” in 2015 and how it affected the position of young migrants in society, researchers have underscored the value of studies challenging one-sided images of migrant youth. This study examines how migrant positions are constituted, negotiated, and related to young people’s well-being. The study was undertaken using an ethnographic approach combined with the theoretical concept of translocational positionality to acknowledge how positions are created through historical and political processes and, at the same time, are context-dependent over time and space and thus contain incongruities. Our findings show how the newly arrived youth used multiple ways to navigate the school’s everyday life and ascribed migrant positions to achieve well-being as illustrated through the distancing, adapting, defense, and the contradictory positions. Based on our findings, we understand the negotiations that occur in forming migrant positions within the school as asymmetric. At the same time, the youths’ diverse and often contradictory positionality showed in various ways the striving for increased agency and well-being.

## Introduction

Migration is one of the social determinants affecting young people’s health [1], and the contribution of migration is recognized in the 2030 Agenda for Sustainable Development. Still, how migration “lingers” and continues to be an active part of young people’s lives in their post-migration period, affecting their everyday navigation and possibilities for well-being, is a somewhat absent perspective in research. Especially in the Nordic context, where this study takes place, there is a lack of research focusing on the well-being of young people with migration experiences.

Children and young people accounted for almost 13 percent, 36 million, of international migrants in 2020 [2]. In 2015, many young people and children moved across borders to Europe, and young people from war-torn countries such as Afghanistan, Syria, and Somalia came to seek protection in Sweden, among other places. The political context in Sweden regarding asylum seekers and refugees in the post-2015 period shifted. Sweden went from opening to closing its borders; a law in 2016 made it difficult for young people to obtain permanent residence permits [3]. Sweden’s previously open and inclusive reception was replaced by migrants adapting to values associated with Sweden and “Swedishness” [4]. Thus, what has come to be known as the “refugee crisis” of 2015 made demands on and, at the same time, challenged European countries and especially Sweden’s image as recipient-friendly and humane [5–7].

Researchers have highlighted how framing the 2015 migration as a “crisis” has led to dichotomized positionings of young people. For example, unaccompanied migrant youths in Sweden are positioned as threats or victims, depending on whether they are considered deserving of protection [6]. Similar findings have been reported in other European countries [8]. Considering this dichotomic positioning, researchers have underscored the value of studies that challenge “the single story” and one-sided images of migrant youth [5, 9]. Above all, there is a lack of studies within the Nordic context exploring newly arrived youths’ positionality from their perspectives.

Against the backdrop of increased youth migration in combination with a more restrictive migration policy in the Nordic countries, it is urgent to study young people’s everyday life and opportunities for health and well-being in the recipient country, hence Sweden in this case. Not least is it important to investigate how young people’s possibilities to negotiate their well-being relate to perceptions of migrants and migratory processes, an area on which there is a lack of research. There are studies that, on the one hand, shed light on how migrant positions are created and, on the other hand, deal with young people’s well-being. Still, there is a lack of studies that explore how migrant positions influence young people’s well-being in everyday life. This article intends to contribute to the knowledge gap about how young people with migration experiences negotiate ascribed positions and the ascribed positions’ relationship to their well-being. We used an ethnographic approach combined with a translocational perspective to capture the complexity of newly arrived young people’s well-being in everyday life in school and to examine how migrant positions are constituted, negotiated, and related to young people’s well-being. By this combination, the study provides a unique insight into the daily life of young people who recently migrated to Sweden, the constitution of migrant positions in school, and how it influences the conditions for young people’s health and well-being. These processes take place in a local context in northern Sweden, affected by the migration policy changes that have taken place in recent decades in the Nordic countries. At the same time, the processes reflect global phenomena also seen in other European countries and elsewhere. Therefore, the study’s findings are relevant and applicable outside the local Swedish context.

## Previous research and theoretical framework

Although there has been a shift in the view of health that started as early as 1946 with the World Health Organization’s broadened perspective on health, biomedical adult-centered approaches continue to be the norm in the study of young migrants’ health [10]. Studies have concentrated on psychiatric symptoms, trauma, and emotional problems linked to the time before migration and the recipient country in the medical and behavioral science research field [11, 12]. The perspective is on individuals and their problems, unilaterally shaping the image of young unaccompanied refugees as a particularly vulnerable category with the risk of pathologizing and othering these young people as a deviant category [11].

These approaches are limited in understanding broader environmental contexts shaping young people’s health [10]. In contrast, this article is placed within a growing field highlighting an inside perspective in the studies of young migrants’ living conditions, including the youths’ narratives, focusing on aspects of the post-migration period and experiences of belonging and inclusion/exclusion [13]. Focusing on children and young people’s migration experiences can be considered a more recent area of research that has emerged at the intersection of youth and migration research, the so-called “childhood turn in migration research” [14].

Outside the health field, still linked to an inside perspective and narrative tradition, an increasing proportion of studies consider young migrants’ conditions in school. School is an integral part of newly arrived youths’ everyday life, and it is a crucial context in their first post-migration period. As the first encounter with society, it is a vital venue for negotiating future opportunities for integration [15]. Studies have shown that school is an essential arena for young migrants’ sense of hope for the future, sense of belonging, and security and structure in everyday life [16–18]. Also, as a crucial social arena, teachers and friends are highlighted as necessary [19, 20].

Findings from studies on Language Introduction (LI), the educational program in Sweden aimed at newly arrived young people, also show segregating patterns, experiences of exclusion, and parallel school life among the newly arrived youths [4, 19, 21, 22]. These findings are also in line with studies showing how young people with a foreign background and youths perceived as “not Swedish” have experienced being positioned as “problematic,” “deviant,” or as “the other” within the Swedish school system [23–25]. This may impact newly arrived young migrants’ future opportunities to form their own lives, self-perceptions, and well-being.

Although more studies in recent years draw attention to young people with experience of migration, there is a lack of research focusing on these youths’ strategies for well-being and capturing everyday micro-processes in place rather than retrospectively, i.e., allowing young people to look back on their experiences in interviews [19]. Also, there is a need for research that broadens the understanding of how health and well-being occur in young people’s lives and thus provide theories that can capture the many complexities surrounding newly arrived young people’s health.

With the ambition to contribute to the growing field emphasizing the perspectives of migrated young people and their living conditions in the study of health and well-being, this study is undertaken by an ethnographic approach combined with a translocational perspective. By this theoretical combination, the study also contributes to the methodological and theoretical development of the field. Overall, this study assumes that young people’s health and well-being are created at the intersection of migration regimes, educational environment, and everyday life. It is based on an overall constructivist perspective on health and well-being, focusing on social, cultural, material, and structural dimensions. From this perspective, health and well-being are created, made possible, and negotiated in daily practices, interpersonal relationships, and the interplay between individual and structure [26, 27]. Health, in this sense, is not about an inner capacity or a purely physical state. Instead, it can be influenced positively and negatively [28], meaning health inequalities are created by societal circumstances and thus changeable [29, 30]. Well-being is about how the newly arrived youths thrive in their everyday lives and how they are able (or not) to achieve their potential [31]. In line with a capability perspective on well-being, we explore the opportunities that the newly arrived young migrants *perceive* they have to live the lives they value the most [32, 33].

To explore the young participants’ well-being through everyday navigation, the study emphasizes social positioning which focuses on how positions are created socially in interaction and narratives, including attributing positions to others and self-positioning [34]. In line with Anthias [35], we chose the concept of position over identity to acknowledge how positionality is created through historical and political processes and, at the same time, is context-dependent over time and space and thus contains incongruities. To capture how positionality is linked to global power relations, we used the concept of translocational positionality [36]. Through a translocal perspective this study explores how young people are positioned in everyday life at a specific school and time. At the same time, positionings extend over time, space, and geographical locations. Furthermore, we study an “everyday reality” at school; still, this reality is rather unique as the young people find themselves in non-ordinary situations.

The perspective of translocality is based on the critical and postcolonial theory where the gaze is turned from studying the subordinate to studying processes that construct “the other” in an institutional, socio- and geopolitical context [37, 38]. The advantage of this perspective in analyses of young people’s health and well-being is that it makes visible the conditions and the social and cultural contexts in which young people’s health, and inequality, are created [27]. Translocality here refers to interconnecting social positions with the sociopolitical and historical processes that shape conditions for health–anchored in a local place [27, 38]. It is a way of bringing together quantities, such as individual and structure, based on the understanding that the individual’s “choice” depends on structural conditions. Lifestyle choices, how an individual “chooses” to live her or his life, and how these “choices” effect opportunities for health depend on local and global perquisites. Thus, examining the contexts that shape young people’s health is crucial, rather than focusing solely on individual lifestyle choices [26, 27].

Following Anthias, this study makes the theoretical presumption that migrant positions are complex and context-dependent, created in everyday social interaction, and exist at the intersection of several power structures, such as age, gender, and race, linking to political and historical processes. Without further developing the categories of age, gender, and race theoretically, these are understood as socially, symbolically, and politically constructed [39]—which does not mean they do not have real effects on people’s lives [40]. On the contrary, this study shows how creating and re-creating categories forms positions important for young people’s well-being.

## Methods and materials

To examine how migrant positions are constituted, negotiated, and related to young people’s well-being, we used an ethnographic approach combining participant observations, qualitative interviews, and informal conversations [41]. The study was conducted with the approval of the Swedish Ethical Review Authority (Dnr 2021–01389).

### Selection of case, participants, and limitations

As described above, school is a crucial arena for newly arrived young people as they have significant adults and peers to relate to and learn about the receiving society. Also, it is an arena where young people spend a large part of their everyday life, consequently affecting their opportunities for well-being in different ways. In addition, Sweden, at the time of the study, after the large influx of refugees in 2015, not only received many migrants, but has also adapted a new ​​migration policy that can affect the health and well-being of young migrants. This is something that can also be related to similar changes in other countries. It was against this background that the selection of case—newly arrived young people’s conditions for health and well-being in the context of a school in Sweden—was made. One of the study’s strengths is that it makes visible the contextual conditions that affect young people’s opportunities for health. By doing so, the study can also better account for contextual differences when compared with other national contexts.

The school where the study took place was located in a medium-sized Swedish city with comparatively few foreign-born people, which was also reflected in the composition of students. In the academic year 2017, when the fieldwork was carried out, around 15% of the students at the school had a foreign background, including the newly arrived students [42]. The participants of this study were officially classified as “newly arrived,” i.e., a young person who has been a resident abroad and has attended school in Sweden for less than four years [43]. They were enrolled in a LI. LI is offered to young people who have recently arrived in Sweden as part of an introductory program for students not qualified for the national program in upper secondary school [44]. At the time of the study, 73 newly arrived students were enrolled in the LI (compared to the approximately 2,500 students who attended the entire upper secondary school). A heterogeneity sampling [45] was used. In communication with the teachers, the first author chose the participants based on collecting material depicting students’ different life situations regarding gender and nationality, whether they were unaccompanied minors or living with family, and their residence status. The participants (N = 10, 16–20 years old) were male (N = 7) and female (N = 3) from Afghanistan, Syria, Kosovo, Somalia, and Ethiopia. Seven were unaccompanied refugees, and three had come with their families. Four stated they had already received residence permits, one living with family had a temporary work permit, and two indicated that they were in the process of seeking asylum. They came from different socio-economic backgrounds and with varying school experiences. The ten young people are counted as the main participants, and the first author had the opportunity to interview them and to regularly follow up their informal conversations.

That there are fewer women than men in the sample reflects everyday life at the school and the fact that the majority were men on LI at the time of the study. The selection thus reflects the empirical material, for example, how a greater focus on the school’s everyday life during lessons was directed towards the boys and the “male role” discussed in the study. In addition, initial thoughts and analyses were discussed with participants and school staff. However, the final results were not discussed with participants, which can be considered a shortcoming. Another limitation is that even though I (first author), talked to many students who reflected on their life situations, I did not have the opportunity to interview individuals from all nations represented in the LI. An advantage of the material is that it comprised information on different lessons with different teachers as well as different locations, enabling the study of varied positionings.

Information about the study was given to the young people and the school staff in writing and orally, and written consent was obtained from participants before the study began. The students were informed that their participation was voluntary, and they could withdraw at any time without explanation. They were also told that field notes from the observations would be recorded in a way that individuals could not be identified and that pseudonyms would be used. All participants’ names and names of places are pseudonyms.

### Observations and interviews

The first author carried out the fieldwork but communicated with the other authors in an ongoing analysis that ran parallel to the data collection, described below in the analysis section. As the first author, I spent 20 entire school days on-site (April–June 2017). Based on an exploratory approach rooted in everyday life, the study started with broader research questions with the purpose of understanding what well-being signifies for young people and what they do to achieve well-being in daily life. During fieldwork on-site, the recurring difference-creating processes and migrant positions became more and more pronounced as processes that formed a large part of the young people’s navigation in everyday school life. Thus, the focus narrowed to how the young people negotiated their well-being in relation to how they were positioned as migrants and on how “boundaries” were drawn between “Swedes” and young people from “other countries.” I studied how “Sweden” and other countries were presented, and how the young people reacted to these representations. I also observed how (ill) health was handled, addressed, and linked to migrant positions. The observations were mainly done during lessons, but I also participated in excursions outside the school premises and spent time with the students on breaks. The field notes from the observations were handwritten and structured as follows [46]: (P) the physical setting and participants. (A) activities, interactions, and conversations: descriptions of “what happens;” (R) reflections: including notes about how I reacted to and reflected on events and what emotions they evoked in me. This record has been used to reflect on ethical aspects and my role as a researcher in relation to the students.

All participants partook in one formal interview that lasted, on average, 40 minutes, followed by several more informal conversations in daily school life. The interviews took place individually within the school and were held in Swedish. The interviews were audio-recorded and transcribed, and the informal conversations were recorded as field notes [41]. The interviews served as a follow-up on the situations and social practices that had shaped the newcomers’ positions; participants’ reasoning about these situations also deepened the observational material. Overall, the interviews aimed to understand how the young people viewed their well-being and space for health in an everyday context. Questions were asked to all participants about how they experienced their life at school and outside and how they experienced their well-being today. I was sensitive to whether they wanted to talk about their past because this varied among the young people. For example, some were very secretive about the relationship with the family that they may have left and broken contact with. However, questions also involved how they grew up and how they looked at their future. This contributed to understanding how the participants experienced and negotiated the newcomer position concerning their well-being.

Finally, fieldwork is a social act where the material is created in the relationship between the researcher, the participants, and the “field.” I believe that several of the youths saw me as a “representative of the majority society,” which was evident, for example, when one participant expressed that I was “a good Swedish contact.” This, in a way, “outsider” position may have enabled some stories and excluded others [47]. The young people sometimes clarified aspects of their life when they assumed that I did not share the same cultural frame of reference, which might not have emerged through an insider position. At the same time, knowledge may have been lost as an (at least imagined) shared cultural frame of reference could have been provided.

### Analysis method

The analysis was characterized by an abductive approach; hence, an ongoing analysis process accompanied the data collection and involved a movement between theory and data [41, 48]. Almost every day after the field observations, the field notes were rewritten; they were compiled into a weekly field report shared and communicated with the third author. In this way, the data was processed and led to more focused analyses of certain aspects, and the field narrowed [46].

After leaving the site, we intensified the analysis following the six phases of thematic analysis [49]. In phase 1, *familiarizing yourself with your data*, I transcribed the interviews. The interviews were read vertically and horizontally, and the field notes were read in parallel to contextualize the interviews. A first textual coding was done in phase 2, *generating initial codes* and comparing patterns and individual nuances, i.e., focusing on one participant at a time and patterns across participants. The textual coding showed categories such as “wanting to fit in,” “feeling misunderstood,” and “feeling divided.” As much consideration was given to consistent statements and practices as to discrepancies in the various material categories [41]. Positionings were done through Phases 3 to 5, *searching for themes*, *reviewing themes*, and *defining and naming themes*, respectively. The themes were discussed, critically examined, and expanded upon by all authors in relation to previous findings. In this communication among the authors, possible interpretations were tested and, thus, empirically validated [46]. The last (sixth) phase, *producing the report*, included the first author revisiting the research questions, reading literature, and re-reading the data. The description of the analytic procedure may look like a linear process. Still, involved back-and-forth analysis between different parts and the whole proceeding using a cyclical movement between the categorized empirical data and analytical concepts derived from the theoretical perspective informing the article. The discussion of the analysis was ongoing until a consensus was reached to ensure the quality of the analysis [45].

## Analysis

The analysis is presented in three parts. The first provides a brief but necessary description of the context, and it explains how the position “newly arrived migrant youth” was constituted and attributed within the school. The second and more comprehensive section analyzes young people’s negotiations and reactions to assigned migrant positions. Finally, in a concluding section, we discuss, based on the young people’s negotiations and reactions to assigned migrant positions, the implications of migrant positions for young people’s health and well-being. The positions should be read as ethnographic typologies [41], i.e., patterns that are framed pedagogically and analytically, making “reality” more comprehensible. However, in everyday life, the boundaries are more fluid and intertwined.

### How the position “newly arrived migrant youth” was constituted within the school

An important starting point for understanding how migrant positions, and especially the position “newly arrived migrant youth,” were constituted within the school is through the meaning of the temporary and uncertain status that the newcomer category indicates. There were many asylum seekers on LI at the time. The prevailing political situation, with deportations as a real possibility along with difficulties in obtaining residence permits created a tense situation at the school. The young people were daily assigned *temporary positions*, such as migrant, refugee, victim of war, and newly arrived student. Students who did not show up for a class or suddenly disappeared may have been temporarily absent or have left the country. Government decisions and processes that concerned the students’ situations and status extended beyond the school grounds and could arise at any time and disrupt life at school. These dislocations [50, 51] interrupted the school day, creating situations when schoolwork stopped, and the focus turned to the young people’s asylum processes or events in their home country—a bombing in Afghanistan, for instance—contributing to constituting the newcomer position as temporary, fragile, and different from other student positions at school.

Consequently, the focus was on the young people’s emotional ill-health. Lesson time was devoted to processing “traumatic experiences” concerning their route to Sweden. However, as previous research has described, there is a disagreement between teachers as to whether it is within their purview to tend to newly arrived youths’ well-being and processes that extend beyond learning, such as contact with a legal guardian, the housing situation, etc. [20]. The lessons and their focus varied with different teachers and in different classrooms at this school. Also, since the upper secondary school reform (GY11), the eligibility requirements for the national programs have been raised. With stricter eligibility requirements in combination with students who came to Sweden at older ages and with a more varied school background, as was the case in this school, there was noticeable relinquishment of duties relating to the students and frustration among teachers. Hence, there were several circumstances that concerned both changes on the educational side and the migration area that affected everyday life at school and the relationship between teachers and students.

Besides their route to Sweden, the youth’s upbringing and background were concerns that were given a lot of attention during class and linked to their emotional ill-health. Thus, discussions about *cultural differences* formed a pattern for understanding how assigned migrant positions were constituted within the school. The newly arrived students’ cultural backgrounds were under constant negotiation and were a recurring element during lessons. Although their “cultures” were presented positively and negatively, they were often based on a static view of culture, with Swedish culture as the norm [24, 25]. Thus, the young people’s imagined cultures and “cultural backgrounds” were portrayed as different in relation to an imagined Swedish culture. The newly arrived position was thereby created regarding “ideas about Swedishness” and the position “Swedish” as a normative social position [40]. Gender roles were often at the center of these discussions.

In these general descriptions and conversations, Sweden was portrayed as modern, developed, and fostering equality. In comparison, the newly arrived students’ home countries were presented as foreign, violent, and poor. Race was an essential category in creating migrant positions that emerged through recurring demarcations. Sweden and Swedishness became the normalized starting point in discussions and were seen as superior to the “other cultures.” Despite the students’ heterogeneous backgrounds, the collective position (speaking to the students as if they were a group sharing a common (hi)story) was a contributing constitutive factor. It was based on *a* unifying factor: the young people were attributed a common position (since they are perceived as not Swedish) outside an imagined Swedish community. Thus, this difference-making practice positions young people as foreign and not yet developed (i.e., different and inferior compared to a normalized Swedish position).

In contrast to the practices that formed the student group collectively, a different pattern was identified in which national representations and the importance of national belonging and citizenship (which created hierarchies between the students) prevailed. The importance of national affiliation was not explicitly articulated but recurred through jokes and subtle twists among students. When I asked a teacher about what I observed, she described a “hack order” among the students and how they usually chose to be with students from the same country. Against this background, the teacher’s strategy to avoid talking about specific countries can be understood. At the same time, these performances created boundaries and hierarchies in classroom conversation that positioned the students in different imagined communities [52]. These hierarchies were created through the actions of teachers and students. The teachers sought support from some students to explain certain phenomena, such as honor killings. The students, in turn, both protected and tried to counter groupings and at the same time reprimanded each other for not following the proper behavior “here.” This created an ongoing struggle between how “here” and “over there” were understood and between ideas of proper and non-proper behavior.

Overall, the newly arrived position was created at the interplay between political progressions and the status of the newly arrived, between temporal and spatial processes that disrupt everyday life at school and conditioned the young people’s health and well-being. At the same time, it created specific locations for the young. These locations, hence attributed migrant positions, have broadly been explained above through positions such as *the temporary position*, *the position as culturally different*, and *the collective position*. We understand these locations, constructed at the interplay of age, gender, and race, as conditions for young people’s identity negotiations and well-being. The next section develops how the young people negotiated these assigned migrant positions.

### How the young people relate to and negotiate newly arrived migrant positions

This section focuses on the young people’s self-positionings, negotiations, and reactions to ascribed migrant positions. The youths used multiple strategies to navigate everyday life in school, as illustrated through the distancing, adapting, defense, and contradictory positions.

#### “Now I am not ‘them’ anymore:”–The distancing position

One of the strategies the young people used to deal with attributed migrant positions was through performances of distancing. The distancing position included acts of dissociation from the home country and associated phenomena, such as a culture of honor or religious attributes like the veil. These topics were actively discussed in class. Nevertheless, the strategy was also about distancing themselves from a way of thinking and being. This position can be understood against the background that the youth have left their home countries for various reasons. For example, Samir described not feeling welcome in his former home country. He has Afghan heritage but has never been to Afghanistan, having grown up with his family as a paperless migrant in Iran. Samir took a critical position toward his former home country in his story, recounting negative experiences. His reflections revolve around his position as an undocumented person and the choice he was forced to make before coming to Sweden: fight or flee. Similarly, Najib described that it was not possible to live in his Afghan village, only to survive. The distancing position can be understood in a broader, temporal, and historical context. Some young people left their home countries due to war, famine, and complex conditions.

The distancing position can be seen in the light of how their home countries and “cultural backgrounds” were depicted in negative and problematic ways at school. Through these notions, positions of superiority and subordination and boundaries between imagined communities and people were constructed. In relation to these discussions, the young people marked distances from national affiliation and negative phenomena they did not want to be associated with. For example, when the students during a lesson discussed corruption and reflected on the degree of corruption in their home countries, one student asked another, “Why do you say ‘them’ and not ‘us’?” The student answered, “Now I am not ‘them’ anymore.” The student marked distance from national attribution; he did not want to be associated with corruption.

Similarly, some young men dissociated when discussing phenomena such as religious extremism, honor killings, and terrorist attacks, stating they had no experience or knowledge of these subjects. However, others who did describe having experience of negative events—for example, witnessing violence—nevertheless distanced themselves from them. These practices should be understood against the focus devoted to the male role in “developed” versus “underdeveloped” countries. Concerning these discussions, dichotomous positions were created. Generally, while men from underdeveloped countries were described as patriarchal and different, Swedish men were portrayed as equal to women: they helped at home, took parental leave, and treated women well [53]. The teachers emphasized that not all Swedish/foreign men fit those roles but still depicted such portrayals as the norm. Through such conversations (different-making practices), the newly arrived male was positioned as deviant due to his patriarchal background. Consequently, the male participants returned to these images and marked their distance during the interviews.

Some actions of distancing, like these, were more apparent than others. Nevertheless, some practices were more inconsistent and showed the tensions contained in the distancing position. The veil, for example, was a recurring subject that became a symbolic boundary between “modern” and “non-modern” countries and between “modern” and “non-modern” women. “In Sweden, women do not wear the veil,” teachers repeated in class. Leah, in conversations, marked her distance from the veil, stating she did not mix with girls who wore the veil. Leah, who came from Syria and lived with her sister, said she liked to hang out in town after school, unlike girls who only stayed home.

Since I often saw Leah with one of the other girls who wore a veil, I asked her about this statement. When asked about it, she answered, laughing, “Yes, but she’s kind.” This reasoning illustrates how the youths’ positionality was situational, contextual, and relational [36]. It seemed important for Leah—toward me—to mark distance from the veil. But her reasoning was not related to her actions. Tanesha, who wore a veil, often ended up in a vulnerable situation in the classroom context, where the meaning of the veil was discussed in her presence. She was told that the veil did not belong in that place; it belonged to another (time and) space.

In these cases, the veil became a symbol that simultaneously marked a Muslim and an undesirable position [54]. At the same time, the girls said they felt pressured by some of the boys to wear a veil. “Otherwise, they would end up in hell,” as Leah said the boys had told them. The discussions about the veil made the boundary between cultural and religious beliefs apparent, with Protestant culture visible as the norm [54]. Demarcations through objects like the veil positioned the young girls as doubly deviant [55]. Thus, it was difficult for them to know how to behave. Both wearing and not wearing a veil could lead to negative consequences. Above all, the veil forced them to “mark their position,” to take a stand or choose a side. Even though, as some of the girls explained, the veil was not an issue, it was just something that some girls wore; it became a charged topic through the classroom discussions.

Overall, the distancing position was constituted in relation to *cultural markers* [56] representing the youths’ home countries and cultural backgrounds, explicitly concerning female and male roles, which in turn contrasted with how they experienced the accepted position in, as they described it, “modern” countries. At the same time, this position pointed to conditions, events, and phenomena that the young people dissociated from for various reasons. It can also be understood as a floating position to the adapting position.

#### “I try to be an ordinary Swedish person”—The adapting position

In addition to distancing oneself from one’s home country, the negative phenomena associated with it, and the gendered positions that the participants described as not accepted here, the participants emphasized the importance of adapting (to Swedish culture). The adapting position can generally be understood as a longing to belong. However, it was remarkable how synonymous *belonging* and *becoming Swedish* were in the youths’ narrations. As explained by Wernesjö [17], narratives about wanting to become “Swedish” can give expression to an aspiration to belong rather than become Swedish.

Najib was one of many who explained well-being as wishing to have “a normal life,” something he initially described during his interview in terms of starting a family and feeling safe. Asked what he feels when thinking about the future, he said:

N: Well, it feels good. You must tell yourself; it’s okay. It will be good /…/ So, I try to be an ordinary Swedish person as normal people do. When you see me, you do not know that person is Swedish. /…/ I want the same (life) as they live. Have more contact with Swedes, have a job, and have a house, the culture, the whole vision.

In Najib’s story, belonging was linked to a Swedish position that comes with, as he described it, certain values, behaviors, lifestyles, and ways of looking at relationships, such as living with a partner outside of marriage. At the same time, he described feeling “different” from Swedes and said he wanted that distance to decrease. He emphasized learning Swedish and meeting more Swedes to reduce the perceived distance and become part of a (Swedish) community. The language was the primary category for positioning oneself as Swedish and thus can be understood as a category that constitutes “ways in which people are ordered and hierarchized” [50]. Najib’s account showed the adapting position captured aspirations to a future desired position constructed to the position “Swedish” as a normative social position [57]. The desired movement from one position to another can be understood as a quest to create a better life, cf. lifelines [58]. As Najib said: “When you live here, you can plan. Keep track of what to do in the future. You can go to school, learn to read, and then read good subjects, and then find good jobs. There (in Afghanistan), you can have no plan for the future because we try to survive.” However, the movement can also be interpreted as a desire to move from a place where one experiences oneself as different from others, “the position of the stranger” [17].

Najib, had a residence permit and thereby the opportunity to plan for the future, but adaptation meant something else for Ali, who was in an asylum process and thus had an uncertain existence. When I talked to him out in the schoolyard, he was just about to take a bus to a nearby municipality—where he currently lived—to arrange travel documents. Ali must make sure that he does not have to move to Kullane. “Now they [the authorities] want me to move to Kullane,” he told me. He got a certificate from the school, but it was not enough. “There is a school there too, they said.” But that he has a job at a nursing home in Ranö this summer helped, he explained. Ali shakes and grits his teeth when we talk, and he looks frozen and stiff even though it is a warm and sunny spring day. We walked across the schoolyard, and he explained that he used to live in Ranö with his friends, he enjoyed himself there, but when he turned 18, he had to move and has since been forced to move several times. He says that moving has become common, something he has adapted to. Thus, for Ali, adaptation means understanding and adapting to the authorities’ rules and decisions to hopefully have a future in Sweden.

As in Ali’s story, the age 18 boundary reappears in conversations in the classroom and the young people’s narratives. Turning 18 meant changing locations, not getting help in the same way as before, for example, through a legal guardian, and that time was running out to finish school and get into “regular” upper secondary school. Age was thus a clear limit, where the transition to adulthood at 18 had extensive consequences for the young. At the same time, the young people were positioned and also positioned themselves as *malleable youth*. Thus, in other situations, their “young” age was presented as an advantage and associated with expectations of change and adaptation. The newly arrived were described as young enough to absorb the “new culture” and thus had a chance to become more than just an “immigrant.” The immigrant position was discussed and presented as a probable but undesirable path. It was a position that the teachers, out of concern for the students, warned against. They described how “Swedes” were not always kind and could maltreat immigrants.

Teachers and students testified to a ranking in the classroom related to notions of cultural superiority and subordination. This pattern emerged through perceptions that some of the newly arrived were already culturally closer to “Western values,” “more adapted,” or “more secularized” when they arrived in Sweden. The adapting position should be understood in light of the school’s mission of “fostering students and clarifying the values that are associated with Swedish society, including democracy, respect for human rights, and gender equality” [20]. The lessons in Swedish focused on Swedish society and culture and which behaviors are accepted in students’ home countries vs. “here,” which led to negotiations on how it is “here” and “over there.” A great deal of focus was on the male role, and for some of the male participants, it was crucial to emphasize having changed since they came to Sweden, especially their way of looking at women and women’s space.

The adapting position includes a marked change or movement. Several of the young people altered between “then and there” and “here and now” positions, reflecting notions of spatial and temporal movements. It also seemed essential to position oneself as adapted in relation to others who had not changed and had not adopted a more “modern” lifestyle. Ali, for example, explained that “Some [students] believe that they can have it in Sweden as in their home countries, for example, having two wives and spouses. It does not work here. You have to be secularized.” The word “secularized” was used in class and by several youths to describe this change linked to religious and masculine positions in their stories. These quotes illustrate how the adapting positions were shaped by the local context and by which (masculine) positions they perceived as *accepted in Sweden*. Admonishing each other and showing other students that they had taken on a new and more equal masculine position was a regular performance, both in participants’ stories and during class. Aaron, coming from a country that was in the minority in the classroom, described this as a “competition.” He told experiences of exclusion in the school and difficulties connecting with Swedes, something he explained was due to cultural differences. Thus, this “competition to be adapted” created conflicts and feelings of exclusion in some students.

As illustrated through the youths’ accounts, the adapting position contained status aspects, such as how well you speak Swedish, how many Swedes you know, and how “secularized” you have become. These status aspects formed the basis of hierarchical orders captured by the adapting position. This position was linked to notions of mobility, illustrating the young people’s striving for a better life, belonging to a community, and acceptance.

#### “There are different ethnic groups; not all ethnic groups are so violent:”–The defense position

Another way of dealing with attributed migrant positions was captured through the defense position. The young people actively protested, objected to, and gave nuance to images of their home countries and associated phenomena. For example, they had different responses to descriptions of themselves as coming from “poor” and “underdeveloped” countries. A common reaction in the classroom was to take an ironic, defensive position. The students joked, for example, that they could study with the lamp off because they came from “poor countries” and were therefore used to simple conditions. Similarly, in conversations, some reported they did not intend to change; one said, “You can move the boy from the culture but not the culture from the boy.”

This position captures strong emotional reactions in the young people, who reported being misjudged. They explained that images of their home countries and backgrounds were incorrect and unfairly portrayed, making them dejected. Rashid explained, “You can say what you want about Afghanistan because no one here knows what it is like,” illustrating an asymmetrical negotiation. Further, Rashid said that although he agreed with certain images of Afghanistan as violent, insecure, and oppressive to women, the group to which he belonged, Hazaras, was not like that. Thus, he defended his ethnic group and explained that not all groups are the same:

Do you know that there are different ethnic groups in Afghanistan? Not all ethnic groups are so violent with women, with their women. There are Pashtuns, Tajiks, Hazaras, and Uzbeks. These four are huge ethnic groups, but there are others as well. There are a lot of people in Afghanistan, do you understand? The Pashtun ethnic groups are a little tougher. With the women. And everyone who is Taliban is Pashtun. They are not Hazaras or Tajiks or Uzbeks.

The importance of relationships with peers emerged through the defense position. How others perceived them mattered. Like Rashid, others expressed concern that peers, primarily Swedish, would view them negatively due to their religious, ethnic, or national affiliation.

In addition, some youths added nuance to these negative images they’d experienced the views Swedes had of refugees and how this reflected on them. Petrus, whose voice was often heard in the classroom and who engaged in discussions with the teachers, explained:

We can find a day where I can tell you everything I have written if you want. I have also written a book about Syria. Do you know why we came? /…/ We are Christians and Muslims. /…/ You know, for Syria, everything came at once. War. IS. Chaos. But really, before the war, it was a rich country. We had good education and opportunities. Right! /…/ Look, I lost my family. They are in Syria. My house. I worked I had a company selling and buying animals. It was my father’s but I who ran it. I also had five dogs, horses, snakes, and birds at home. But then, when you tell a Swede, they did not think we had dogs. I look into their eyes when I say I had five dogs. They say, huh? They’re looking at me. Did you have five dogs? Can you get dogs in Syria? I get very disappointed when I get this, so it’s a lot of prejudice.

Petrus actively opposed teachers’ claims when he had different knowledge or experience. Through this practice, the teachers’ knowledge position came to be challenged. Petrus worked toward changing his home country’s image as poor and associated with refugees, an ambition that extended beyond the school context. He described having written an article published in a Swedish news magazine. He explained that Sweden is a large country on the surface but with a small population that thus needs refugees. However, his statements can be understood in terms of deservingness [6]—he did not see a given place in the country and had to prove he deserved one.

The defensive position captured the young people’s experiences of being subordinate, which forced them to adopt protective counterstrategies. At the same time, their actions show how they strived for well-being. They did not accept this position but acted in various ways to change negative images and increase their agency.

#### “They think I’m happy here in Sweden, that I can go out, but that is not true:”–The contradictory position

As mentioned, the youth had multiple strategies for navigating everyday life. Their self-positionings had to do with “who they were” (or how they acted in school) and with a series of dislocations, processes that stretched across time and space. Conversations with the young people made clear that their positions were negotiated in both temporal and transnational spaces, and the relations with their “home countries” were complex. For example, Hamed, who lived with his family, described feeling dispirited when thinking about the situation in his former home country. At the time of the interview, there had been a bombing in Kabul, and Hamed said that he had lost close relatives. He described the situation in Afghanistan as a disaster and said his family was worried about what would happen if they were not granted residence permits. Hamed dreamed of staying in Sweden, saying, “If it works, it will be great. We will be free, and we can do anything in our future.” Like Hamed, several of the youths described how they lived in their home countries in their thoughts, how they followed the situations there and cared about their loved ones. But they also tried to move both physically and mentally to Sweden, which they saw as creating peace in their minds and opportunities ahead.

Although most locations contain contradictions [56], certain situations and practices more clearly showed the fragmentary and incomplete nature of the youths’ negotiations around identity, belonging, and well-being. For example, the negotiations of another student, Besa, centered on her position as a female. She described having limited agency in relation to (men such as) her brothers and father. But in relation to her female friends at home, whom she talked to almost daily and who believed that she now lived in freedom in Sweden, she occupied a contradictory position. Anthias [59], describes a “contradictory social location transnationally” as a negotiated position that changes in different transnational spaces and where the position, like Besa’s female position, can vary in relation to others. Besa struggled with what she described as choices she had no control over herself. The move to Sweden created expectations for a more central agency and more freedom, but it turned out the other way around, affecting her everyday well-being. The move to Sweden to live with her father and brothers had led to her being more controlled than when she lived with her mother in Kosovo.

Interviewer: You live with your family.

Besa: Yes.

I: Mm, and how is it?

B: It’s a little hard.

I: Okay.

B: Because my dad is a little strict. And my brothers.

I: Mm. In what way are they strict?

B: They do not think I should have a boyfriend. They kind of want me just to stay home.

I: Okay. What do you think about it?

B: I think that’s the worst thing they do. They must be a little bit… so *they must change* when they come here. They do not [whisper].

I: Did you think they were strict even when you lived in your home country?

B: But Dad was not home then. He lived here in Sweden.

I: Well, he lived in Sweden.

B: He’s been living here for about five years.

I: Yeah, okay.

B: But he used to come every three or six months.

I: Okay, mm. What was life like when you lived there then, in Kosovo, huh?

B: Well, yes, I studied, like going from (name of the city) to (name of the city). I did not have much time to be with my brothers and stuff.

I: Okay.

B: But still, I had that feeling that I was not allowed to do anything, have a boyfriend, or go out.

Besa’s negotiations of agency highlight what Anthias [36] described as “positionality faced by those who are at the interplay of a range of locations and dislocations in relation to gender, ethnicity, national belonging, class and racialization.” Besa believed her struggles for agency depended on whether her father and brothers would change now that they lived in Sweden. Visible in Besa’s narration and in other participants’ stories are preconceptions they held about Sweden before arriving. In line with previous studies (cf. [6]), Sweden is described in the youths’ narrations in terms of freedom and the land of opportunities. These images of Sweden reappeared in the participants’ narratives and were constructed in the teachers’ stories about Sweden. Their positions were negotiated concerning these national representations and within these trans- and dislocational spaces. At the same time, as for Besa, this position of freedom appears not fully accessible (or conditional) to the young newcomers. Besa did not describe herself as in an inferior position. She explained that she already had a job and had plans to manage herself in the future. Still, she and other newly arrived young migrants struggled with conflicting feelings when trying to understand their place(s).

The contradictory position illustrates that school is one arena among many. The positionality of the young migrants is diverse and often contradictory, and it extends beyond the school grounds and over time and space, through parallel processes. These processes include negotiations and renegotiations about identity and belonging in relation to family and friends in different places. At the same time, the school is an institution that reflects societal processes and is an important arena for this group of young people.

### Implications of migrant positions for young people’s opportunities for health and well-being–a concluding discussion

This study examines how migrant positions are constituted, negotiated, and related to young people’s well-being. We have described how the newly arrived youths in a school in Sweden were positioned in a way that reflected a global hierarchical economic, political, and essentialist cultural order. A conclusion is that the daily life of this specific school was organized by an invariable translocal hierarchical order, something also described in several other studies [23–25]. The school (teachers) celebrated and struggled to combine democratic ideals such as equality, gender equality, and anti-racism. Consequently, they distanced themselves from the attributed situations in the newly arrived youths’ home countries—and expected the same from the students—while engaging in their integration into Swedish society. Through this distancing, the school reproduced what has been referred to as Swedish exceptionalism, a unique national self-image in which Sweden is characterized by far-reaching democracy and gender equality [60]. However, this reproduction of Swedish exceptionalism, with its celebrated democratic and inclusive values, paradoxically risks othering newly arrived young people as a deviant category (cf. [53]). Also, as shown in this study, it risks narrowing their possibilities to negotiate their well-being.

Previous studies describe young people who recently arrived in Sweden, especially unaccompanied minors, with limited or conditional space to negotiate their positions and belongings [6, 17, 61]. The school can be understood as an institution that reflects societal beliefs and which, at the same time, tries to deal with this in the school’s everyday life, based on the idea of contributing to the integration of young people into society [20, 27]. Findings from this study show, in line with these previous studies, how significant attention was directed towards the youths and in particular men’s gender and culture in the school’s daily life. This attention can be understood against an ongoing societal debate about young migrant men as a potential threat and where their cultural backgrounds have been in focus [61]. Stereotyped perceptions of migrants are not unique to the Swedish context; hence, similar discourses about young men who migrate can also be seen in other countries [8]. The findings of this study point to the importance of elucidating constructions of migrant positions in health efforts to understand and work with the processes that affect young people’s opportunities for health and well-being in everyday life in the short and long term.

The daily processes explored in this study also make visible the need of a broadened understanding of what health is and how health occurs in young people’s everyday lives. It includes moving beyond a static understanding of young people’s health as a state of “complete well-being” to an interpretation of the concept as an ongoing process (cf. [62, 63]. That involves, on the one hand, opportunities and the ability to deal with different situations; on the other hand, consideration of the power processes that limit individuals’ agency in daily life. How the young people in this study handle being positioned as migrants can be understood as a part of a quest for well-being. It is regarding the sometimes limiting positions–where young people are reduced to their ethnic/national/cultural background–that the newly arrived young people in this study negotiated their well-being. Hence, understanding the creation of migrant positions in daily life is vital for understanding the creation of health, ill-health, and inequality. Related to these limiting positions, the findings from this study show how race constructions are significant in the everyday social order of the school. These constructions represent power processes that limit individuals’ agency in daily life. In line with Schmauch [64], the findings show how racist power structures are recreated daily in the tension between Swedishness and being positioned as non-Swedish. Seen outside the Swedish context, this concerns processes where young people are positioned as inside or outside the (imagined) majority society, processes which must be understood as dynamic and changing and which can enable or limit young people’s inclusion and well-being. Thus, expressions and experiences of everyday racism are essential to work with in school to counteract unequal health. Everyday racism has been described as dynamic, changeable, and elusive [65, 66]. While the young people in this study created close relationships with what they described as important adults at school, such as the teachers, it was at the same time with these important adults that they negotiated their space and their place through the above-described difference-making practices that took place in everyday life.

It was evident, however, that the young people were not passive, they act to increase agency and well-being. They developed multiple strategies for achieving well-being in everyday life by negotiating the ascribed notions of migrants and managing a temporary position and the regulations outside the young people’s and the school’s control. By navigating between a distancing position, an adapting position, a defense position, and a contradictory position, the translocal hierarchical order was both accepted and contested without being either *totally* accepted or overturned. Instead, the young migrants’ negotiations and resistance in ongoing school life could be understood as a temporary repositioning, making well-established and attributed forms of positioning unstable and livable.

The findings in this study suggest that young people’s well-being is dependent on how they are positioned and how they negotiate these positions that are created at the intersection of migration regimes, educational environment, and everyday life. The young people’s reactions and narratives show that, for instance, distancing themselves or adapting constitutes self-perceptions and belonging as vital for their well-being. Thus, we argue in line with Smith et al. [10] that the biomedical adult-centered approaches need to be accompanied by an analysis of the young people’s everyday life and conditions to fully understand the components and processes of health and well-being.

Further, the findings from this study shed light on processes that can constrain young people’s well-being and that need to be considered in health promotion initiatives. For example, several studies, including this one, have shown that the Swedish school (re)produces difference [21, 25, 67]. These findings indicate that the school tends to “get stuck” in difference-making practices and dichotomous thinking when it comes to young people assumed to have a “different cultural background” than “native (Swedish)” youths. The findings from this study show how the youths were linked to problematic places, and thus forced to defend and distance themselves from their home countries and “cultural” backgrounds—which *can* contain negative aspects as well as positive memories, capabilities, experiences, and not least ongoing transnational relationships essential for their well-being. We believe that an excessive focus on difference is not only “doing difference” in practice. It is also an obstacle to newly arrived young people’s opportunities to thrive in their everyday lives and achieve their potential (cf. [31]). There is also a risk that the assigned positions (such as the temporary and culturally different) will be strengthened (despite the school’s democratic ambitions) as right-wing populist, racist and nationalist political currents gain more and more attention, as is the case in the Nordic, but also other, countries e.g., [3].

Thus, other ways of promoting young people’s health and well-being are needed. We propose that the school can apply and benefit from a translocal approach to promoting the well-being of young people. Such an approach takes its starting point in the local context and the young people’s perspectives and situations and at the same time considers sociopolitical and historical processes as well as transnational relationships important for young people’s well-being.
